# Progressive Supranuclear Palsy (PSP): An Orthoptic Assessment

**DOI:** 10.22599/bioj.505

**Published:** 2026-04-10

**Authors:** Dominic Burdon, Stephen Mullin, Christopher Harris

**Affiliations:** 1 Royal Eye Infirmary, University Hospitals Plymouth, UK; 2 Neurology department, University Hospitals Plymouth, UK

**Keywords:** Atypical Parkinsonian disorder, neurology, orthoptic assessment, progressive supranuclear palsy (PSP), vertical supranuclear gaze palsy

## Abstract

**Aim:**

Progressive supranuclear palsy (PSP) is an atypical Parkinsonian disorder which, like other atypical Parkinsonian disorders, displays neurological motor and oculomotor signs at different stages along the disease course. The overlap in presenting signs between the disorders presents a diagnostic challenge for the clinician, particularly early on. Here we audit the role of orthoptic oculomotor and eye-tracker assessment for atypical Parkinsonian patients.

**Method:**

A retrospective analysis was conducted for 26 patients with atypical Parkinsonian signs, referred to orthoptics for oculomotor assessment. Orthoptic diagnoses were made after reviewing oculomotor range, doll’s head manoeuvre, saccadic velocity, vergence, eyelid signs and fixation results. The orthoptic diagnoses were compared with the final neurology/neurosurgery diagnosis for consistency.

**Results:**

Of the 19/26 cases who were diagnosed with ‘suspected PSP’ after orthoptic assessment, 14/19 (73.68%) had a final diagnosis of ‘suspected PSP’ by neurology/neurosurgery. In 5/19 cases (26.32%) the patients demonstrated PSP-like signs in orthoptics but later received alternative diagnoses. The orthoptic diagnosis was consistent with the final neurology/neurosurgery diagnosis for ‘suspected non-PSP’ in all seven cases.

**Conclusion:**

Suspected diagnoses after orthoptic assessment were consistent with the final neurology/neurosurgery department diagnoses in 80.77% of patients. We conclude that early orthoptic oculomotor and eye tracker assessment of atypical Parkinsonian patients is clinically effective for ruling PSP in or out of the clinical picture.

## Introduction

Atypical Parkinsonian disorders are a group of neurodegenerative conditions that can resemble idiopathic Parkinson’s disease (PD) in their early stages but later present with characteristic multisystem signs. Multiple system atrophy (MSA) and dementia with Lewy bodies (DLB) are atypical Parkinsonian disorders which, like PD, are associated with abnormal intracellular accumulation of α-synuclein (formed into Lewy bodies in PD and DLB), leading to neural and glial cell death (Srivanitchapoom *et al*., 2018). Corticobasal syndrome (CBS) and progressive supranuclear palsy (PSP) are atypical Parkinsonian tauopathy disorders where the protein ‘tau’ causes neural and glial cell death through neurofibrillary tangles (Dickson *et al*., 2010).

PSP, first described in 1964 (Steele *et al*., 1964), presents with neurological motor and oculomotor signs. The classic presentation is called ‘Richardson’s disease’ (Williams *et al*., 2008), but there are now 7 other named PSP phenotypes where presenting signs vary: PSP with predominant Parkinsonism (PSP-P), PSP with pure akinesia with gait freezing (PSP-PAGF), PSP with corticobasal syndrome (PSP-CBS), PSP with predominant speech and/or language dysfunction (PSP-AOS and PSP-PNFA), PSP with predominant frontotemporal dysfunction (PSP-FTD), PSP with cerebellar ataxia (PSP-C) and PSP with primary lateral sclerosis (PSP-PLS) (Respondek and Höglinger, 2016).

Age-adjusted prevalence for PSP is estimated at 6.4 per 100,000 (Schrag *et al*., 1999). PD has an age-adjusted population of 139 per 100,000 (Porter *et al*., 2006). The challenge of differentiating PSP from PD and other atypical Parkinsonian disorders lies with there being no known testable biomarkers for PSP. This means in vivo diagnosis relies upon careful clinical assessment, and a diagnosis of ‘definite PSP’ can only be confirmed at post-mortem (Höglinger *et al*., 2017).

Identifying oculomotor signs in atypical Parkinsonian disorder patients is key to differentiating PSP from other atypical Parkinsonian disorders and PD. Oculomotor abnormalities in PD commonly include impaired smooth pursuit, hypometric horizontal and vertical saccades, but normal saccadic velocity (Pinkhardt *et al*., 2012). Other signs include abnormal square wave jerks and impaired vergence (Leigh and Zee, 2015). This is different to the oculomotor presentation of PSP. These signs include vertical supranuclear gaze palsy (VSGP), slow velocity vertical saccades (SVVS), apraxia of eyelid opening, blepharospasm and reduced blink rate (Chen *et al*., 2010; Leigh and Zee, 2015; Phokaewvarangkul and Bhidayasiri, 2019). Evidence demonstrates that VSGP and SVVS are highly specific and sensitive signs for PSP (Respondek *et al*., 2017). Vergence and fixation are also commonly affected in PSP (Leigh and Zee, 2015). Patient symptoms reported are diplopia/blurred vision (61%) and photophobia (43%) (Nath *et al*., 2003). These PSP signs and symptoms require careful clinical investigation in an orthoptic clinic, with the aid of eye-tracking facility for assessment of subtle early oculomotor signs (Clark *et al*., 2019).

Other atypical Parkinsonian disorders also display oculomotor signs. Zhou et al. (2022) discovered oculomotor dysfunction in 93.3% of their MSA cohort and suggest that it can be identified early in the disease course. MSA oculomotor signs present as cerebellar or vestibulo-cerebellar signs: gaze-evoked nystagmus, positional downbeat nystagmus, rebound nystagmus, impaired pursuit, saccadic hypometria (horizontal and vertical) and poor vestibulo-ocular reflex suppression (Anderson *et al*., 2008). Abnormal square wave jerks are commonly present and occasionally mild vertical supranuclear gaze palsy, representing two signs which crossover with PSP (Anderson *et al*., 2008). Oculomotor assessment can help differentiate between MSA-C and MSA-P, as the MSA-C phenotype presents with lower pursuit gain, lower OKN gain and higher incidence of saccadic hypermetria (Zhou *et al*., 2022).

In dementia with Lewy bodies (DLB), horizontal and vertical saccade latency, accuracy, peak velocity and anti-saccades are commonly affected (Kapoula *et al*., 2010); this varies from PSP, where vertical saccades are primarily affected. Case reports indicate DLB can present with a gaze palsy, but both horizontal and vertical positions are reportedly affected (Fearnley *et al*., 1991; Lewis and Gawel, 1990).

There is overlap with oculomotor presentations in PSP and CBS. Vertical supranuclear gaze palsy has been documented as being present in up to 59% of late-stage CBS cases but becomes less common in early stages when compared to PSP (Kassavetis *et al*., 2022; Rinne *et al*., 1994). Rinne et al. (1994) discovered that 20% of their CBS cohort had apraxia of eyelid opening. Rarely, CBS may present with slow-velocity vertical saccades but less severely and later in onset when compared to PSP (Rivaud-Pechoux *et al*., 2000). Errors with anti-saccades and increased saccadic latency commonly affect patients with CBS (Rivaud-Pechoux *et al*., 2000).

The crossover of oculomotor signs in atypical Parkinsonian disorder patients presents a diagnostic challenge, and failure to offer a suspected diagnosis is not uncommon, particularly earlier in the disease course. Here, we present a retrospective analysis of 26 patients with suspected atypical Parkinsonian disorder. These patients were referred from our local neurology department for oculomotor assessment to help rule PSP in or out of the clinical picture. All patients underwent an orthoptic clinical assessment designed to identify PSP oculomotor signs (see PSP orthoptic assessment). Using data collected from the test results, we compare orthoptic diagnoses (suspected PSP or suspected non-PSP) with final in vivo neurology/neurosurgery diagnoses.

## Methods

For this audit a retrospective analysis of records was conducted. The cases analysed were suspected to have atypical Parkinsonian disorder and hence referred from neurology or neurosurgery to the orthoptic department for oculomotor assessment. An atypical Parkinsonian disorder was suspected in neurology/neurosurgery where patients were inconsistent with the early natural history of PD. The most common symptoms include early falls/postural instability, diplopia or oculomotor abnormalities, cerebellar signs, early cognitive involvement, cortical sensory signs, apraxia, failure to respond to levodopa therapy, urinary retention, and/or early autonomic dysfunction.

The following inclusion criteria were used:

1. Referred to orthoptics with a suspected atypical Parkinsonian disorder.
2. PSP orthoptic assessment conducted at the first orthoptic visit.

Data collected for this audit included PSP orthoptic assessment results and final neurology diagnosis information. This data was collected from patients’ notes in Medisoft electronic records, Medisight electronic records, ophthalmology paper records and general hospital paper records. Data was collected for records between August 2019 and March 2024. This data was pseudonymised since the following patient identifiers were removed: the patient’s name, date of birth, address, hospital number, and NHS number. Ethics for use of the clinical data were discussed with the Trust’s research and development team prior to commencement of data retrieval. Access to the data was granted (by the trust) without the need for ethical approval, as this was a retrospective analysis with all patient identifiers removed.

### PSP Orthoptic Assessment

The PSP orthoptic assessment in these cases included tests to identify the well-documented oculomotor signs of PSP (Chen *et al*., 2010; Leigh and Zee, 2015; Höglinger *et al*., 2017; Phokaewvarangkul and Bhidayasiri, 2019).

#### Ocular motor range

Ocular motor range was tested to identify vertical supranuclear gaze palsy (VSGP) (Leigh and Zee, 2015). Vertical gaze palsy (VGP) presented itself as either an upgaze palsy, a downgaze palsy, or a combination of both. A greater than –1 vertical limitation was recorded as a ‘positive’ result for VGP. Where the vertical range was full, a ‘negative’ result was recorded. A –1 upgaze restriction was recorded as an ‘unconfirmed’ result.

#### Vestibulo-ocular reflex

If gaze restrictions were discovered during ocular motor range testing, the vestibulo-ocular reflex (VOR) was examined using the doll’s head manoeuvre. Where there was improvement in vertical ocular motor range on VOR testing, a ‘positive’ result for VSGP was recorded. Where there was no improvement in vertical range on VOR, a ‘negative’ result for VSGP was recorded but a vertical gaze palsy (VGP) was diagnosed. In cases where no VOR test could be performed, an ‘unconfirmed’ test result was recorded. In cases where it was not needed to be performed (due to there being no vertical gaze restrictions), ‘n/a’ was recorded.

#### Saccades

We tested saccades vertically and horizontally in free space and using an eye tracker to identify slow-velocity vertical saccades (SVVS). Here we used the Eyelink 1000 infrared eye tracker. SVVS were diagnosed clinically where a patient had obvious glissadic (reducing velocity approaching the end of the saccade) and multiple hypometric saccades vertically compared to horizontally. This was made clearer by analysing the eye tracker waveform.

#### Convergence

We tested convergence in free space and with eye tracking by bringing a fixation target to the nose and estimating the ‘break point’ distance. On the eye tracker, the fixation and ‘break point’ were objectively assessed by analysing the waveform, whereas this was assessed in free space by observing the patient’s eyes. The result was recorded depending on whether the ‘break point’ was closer or further than 10 cm from the face. Where the ‘break point’ was further than 10 cm, or where there was no vergence (convergence or divergence), a ‘positive’ result was recorded for impaired vergence. Where the break point was 10 cm or closer, a ‘negative’ result was recorded.

#### Eyelid function

Eyelid function was tested by asking the patient to close and then open their eyelids. A diagnosis of eye lid opening apraxia was given where the patient showed any incapacity to open their eyes quickly after closing them. We tested the patients’ blink rate by observing them during a case history or eye tracker assessment. Where there were fewer than five blinks per minute, a reduced blink rate was diagnosed.

#### Fixation

We used an eye tracker to confirm if macro square wave jerks (SWJ) or frequent SWJ were present in our cases. A positive result was recorded where the SWJs were greater than 1° (macro SWJ), where the SWJs were more frequent than 10 per minute (frequent SWJ) or where there was a combination of both.

#### Diagnosis

All patients were referred for oculomotor assessment on the basis that they had suspected atypical Parkinsonian disorder. A ‘suspected PSP’ diagnosis was given where the PSP orthoptic assessment identified vertical supranuclear gaze palsy (VSGP) or slow velocity vertical saccades (SVVS) in isolation, or where two or more PSP ocular signs of eyelid issue (eyelid opening apraxia or reduced blink rate), impaired vergence or abnormal square wave jerks were present (Chen *et al*., 2010; Leigh and Zee, 2015; Phokaewvarangkul and Bhidayasiri, 2019). If neither of these two criteria were met, a ‘suspected non-PSP’ diagnosis was given. The cases were subsequently diagnosed with either ‘suspected PSP’ or ‘suspected non-PSP’ following orthoptic assessment.

The most recent diagnosis from the patient’s neurologist/neurosurgeon was recorded for comparison. The final neurology/neurosurgeon diagnosis was based on clinical presentations and the Movement Disorder Society criteria (Höglinger *et al*., 2017). These criteria are the consensus guidelines and consider both oculomotor and non-oculomotor signs.

## Cases

Twenty-six cases were audited, of which 19 were male and 7 were female. All cases were between 48 and 81 years old at the time of the presentation of disease signs. The results are displayed in subgroups for ‘Suspected PSP’ patients (Table 1) and ‘Suspected Non-PSP’ cases (Table 2) relating to the orthoptic diagnosis. Results displayed include oculomotor abnormalities, eyelid signs, fixation results and diagnoses. Here, we compare the consistency of orthoptic diagnoses with neurology/neurosurgery diagnoses.

**Table 1:** Oculomotor signs and diagnoses for the ‘suspected PSP’ cohort.


CASE	OCULOMOTOR ABNORMALITY				EYELID SIGNS		FIXATION	DIAGNOSIS	
			
	VERTICAL GAZE PALSY (VGP)	VERTICAL SUPRANUCLEAR GAZE PALSY (VSGP)	SLOW VELOCITY VERTICAL SACCADES	IMPAIRED VERGENCE	EYELID OPENING APRAXIA	REDUCED BLINK RATE	MACRO OR FREQUENT SQUARE WAVE JERKS	SUSPECTED ORTHOPTIC DIAGNOSIS	SUSPECTED FINAL NEUROLOGY/NEUROSURGERY DIAGNOSIS

1	(++)	(--)	(--)	(++)	(++)	(--)	(++)	PSP	PSP

2	(-)	(-)	(--)	(++)	(--)	(--)	(++)	PSP	MSA-P

3	(++)	(++)	(++)	(++)	(++)	(-)	(--)	PSP	PSP

4	(++)	(++)	(++)	(++)	(++)	(--)	(-)	PSP	PSP

5	(-)	(-)	(++)	(-)	(++)	(--)	(--)	PSP	PSP

6	(--)	n/a	(++)	(++)	(--)	(++)	(--)	PSP	PSP

7	(-)	(-)	(++)	(++)	(--)	(++)	(--)	PSP + Hydrocephalus signs suggestive of 3rd and 4th Ventricle involvement	Normal Pressure Hydrocephalus with possible Parkinsonism

8	(++)	(++)	(-)	(++)	(++)	(++)	(--)	PSP	PSP

9	(++)	(++)	(++)	(++)	(++)	(--)	(++)	PSP	PSP

10	(-)	(-)	(++)	(++)	(--)	(++)	(--)	PSP	PSP

11	(-)	(-)	(++)	(--)	(--)	(++)	(--)	PSP	PSP

12	(++)	(-)	(--)	(++)	(++)	(++)	(--)	PSP	PSP

13	(++)	(++)	(--)	(++)	(--)	(++)	(--)	PSP	PSP

14	(-)	(-)	(++)	(--)	(--)	(--)	(--)	PSP	PSP

15	(++)	(-)	(++)	(++)	(++)	(--)	(--)	PSP	PSP

16	(--)	n/a	(++)	(++)	(--)	(++)	(--)	PSP	PSP

17	(--)	n/a	(--)	(--)	(++)	(--)	(++)	PSP	CBS

18	(++)	(-)	(++)	(++)	(--)	(--)	(--)	PSP	IgLON5 encephalopathy

19	(++)	(++)	(++)	(++)	(++)	(--)	(++)	PSP	CBS


For test results: -- = Negative, - = Unconfirmed, ++ = Positive, n/a = Not Applicable.

**Table 2:** Oculomotor signs and diagnoses for the ‘suspected non-PSP’ cohort.


CASE	OCULOMOTOR ABNORMALITY				EYELID SIGNS		FIXATION	DIAGNOSIS	
			
	VERTICAL GAZE PALSY (VGP)	VERTICAL SUPRANUCLEAR GAZE PALSY (VSGP)	SLOW VELOCITY VERTICAL SACCADES	IMPAIRED VERGENCE	EYELID OPENING APRAXIA	REDUCED BLINK RATE	MACRO OR FREQUENT SQUARE WAVE JERKS	SUSPECTED ORTHOPTIC DIAGNOSIS	SUSPECTED FINAL NEUROLOGY/NEUROSURGERY DIAGNOSIS

1	(--)	n/a	(--)	(--)	(--)	(--)	(--)	non-PSP	Primary progressive gait freezing

2	(--)	n/a	(--)	(--)	(--)	(++)	(--)	non-PSP	Idiopathic Parkinson’s disease

3	(--)	n/a	(--)	(-)	(--)	(--)	(--)	non-PSP	MSA-P

4	(--)	n/a	(-)	(++)	(--)	(--)	(--)	non-PSP	MSA-C

5	(-)	(--)	(-)	(--)	(--)	(--)	(--)	non-PSP	Normal Pressure Hydrocephalus

6	(--)	n/a	(--)	(--)	(--)	(--)	(--)	non-PSP	Frontotemporal Dementia with Parkinsonism

7	(--)	n/a	(--)	(--)		(--)	(--)	non-PSP	Idiopathic Parkinson’s disease


For test results: -- = Negative, - = Unconfirmed, ++ = Positive, n/a = Not Applicable.

Of the 19 cases who were diagnosed with ‘suspected PSP’ after orthoptic assessment, 14/19 (73.68%) had a final diagnosis of ‘probable PSP’ by neurology/neurosurgery. 5/19 cases (26.32%) demonstrated PSP-like signs in orthoptics but later received alternative diagnoses. These alternative diagnoses included MSA-P, normal pressure hydrocephalus with possible parkinsonism, IgLON5 encephalopathy, and CBS (two cases).

Where the orthoptic diagnosis was ‘suspected non-PSP’ (Table 2), the orthoptic assessment was consistent with the final neurology/neurosurgery diagnosis of ‘suspected non-PSP’ in all 7 cases. The final neurology/neurosurgery diagnoses in these cases were primary progressive gait freezing, idiopathic PD (two cases), MSA-P, MSA-C, normal pressure hydrocephalus, and frontotemporal dementia with Parkinsonism.

Overall, the orthoptic diagnosis was consistent with the final neurology/neurosurgery diagnosis for either ‘Suspected PSP’ or ‘Suspected Non-PSP’ in 21/26 (80.77%) of patients, with 5/26 (19.23%) inconsistent diagnoses.

## Discussion

In this retrospective analysis the results demonstrate orthoptic assessment is clinically effective for identifying PSP oculomotor signs, as orthoptic diagnoses were consistent with neurology/neurosurgery diagnoses in 21/26 (80.77%) cases. The other 5/26 (19.23%) cases showed PSP-like signs, but alternative diagnoses were eventually made. One of the alternative diagnoses included IgLON5 encephalopathy; this is a condition which can mimic signs of PSP (Macher *et al*., 2021). These alternative diagnoses demonstrate the challenge clinicians face with overlapping Parkinsonism signs in atypical Parkinsonian disorders (Srivanitchapoom *et al*., 2018). Orthoptists are specialists for investigating, diagnosing and managing both developmental and acquired oculomotor and binocularity defects. Orthoptists can use these skills to identify subtle early PSP oculomotor signs, especially where eye tracking is utilised. We recommend orthoptic assessment be conducted for all patients with atypical Parkinsonian signs. This is to aid earlier and more reliable differential diagnosis by ruling PSP in or out of the clinical picture.

During the orthoptic examination, signs of PSP can be recognised from the point of bringing the patient into the clinic room. For example, taking note of the patient’s response and reaction time to being called (indicating bradyphrenia if slow), gait, balance and visual-spatial awareness can all give useful clues. Once the patient is seated, the orthoptist can observe the patient as a case history is conducted, taking note of their communication ability: speech volume, word finding, dysphonic speech or any palilalia/echolalia. Additionally, the orthopist should observe the patient’s facial features for any signs of reduced facial expression, decreased blinking ability, or overactivity of the frontalis muscle.

A full case history for all new patients with suspected atypical Parkinsonian disorder should be performed to understand the timeline of presentation for any neurological and oculomotor signs/symptoms. The orthoptist takes note of any ocular complaints of struggling to move their eyes or eyelids. Additionally, the orthopist should note any reported difficulties with reading caused by diplopia or blurred vision (61%), photophobia (43%) (Nath *et al*., 2003), or confusion of images where two images appear superimposed in the same location (Ansons and Davis, 2014), which may be related to a vergence issue. Difficulty with reading without ocular symptoms could be due to aphasia or bradyphrenia.

Possible general motor signs reported could include postural instability, akinesia, cognitive dysfunction and bulbar dysfunction (Höglinger *et al*., 2017). Bulbar dysfunction may involve dysarthria, dysphagia or drooling. Cognitive impairment could involve signs of apathy, depression or personality change (Nath *et al*., 2003). These cognitive issues may cause the patient to not report the symptoms they are experiencing, further highlighting the need for an exhaustive case history with both the patient and accompanying relative. Accompanying relatives can provide useful background history.

PSP commonly disrupts supranuclear pathways equally in both hemispheres and affects the brainstem inferiorly as the disease progresses (Chen *et al*., 2010). For this reason, vertical and vergence oculomotor systems in the midbrain are affected earlier than horizontal and vestibular oculomotor systems in the pons or medulla (Phokaewvarangkul and Bhidayasiri, 2019). As the disease becomes late stage, the oculomotor nuclei can become affected, disrupting all supranuclear pathways; this would present as a nuclear gaze palsy.

The limited vertical range in vertical supranuclear gaze palsy (VSGP) is typically symmetrical between each eye and will present after the slow-velocity vertical saccades (SVVS). A –1 upgaze limitation was recorded as an ‘unconfirmed’ result here. This is because a physiological limitation of upgaze is not uncommon in elderly patients due to the ageing process of the extraocular muscles. Specifically, the horizontal rectus muscles cause inferior displacement, increasing depressor action and limiting elevator action (Clark and Demer, 2002). As this is muscular pathology, the gaze limitation will remain the same regardless of which supranuclear oculomotor system is tested, enabling the orthoptist to differentiate this from a VSGP.

Improved vertical range during the doll’s head manoeuvre represents the ‘supranuclear’ aspect of the palsy. This is because there is abnormal innervation to the oculomotor nuclei during voluntary gaze (vertical gaze palsy) but preserved innervation to the nuclei during reflex gaze (normal VOR) (Pinhas *et al*., 1978). The doll’s head manoeuvre can be difficult due to axial rigidity. Instead, forcing a forward/backward body movement can sometimes be more revealing.

Vertical saccadic velocity is impaired in PSP when compared to normal subjects, with vertical saccade velocity being significantly slower compared to horizontal saccades (Chen *et al*., 2010). Even in the absence of other oculomotor signs, this can be an early indicator of PSP (Phokaewvarangkul and Bhidayasiri, 2019). The saccades are typically conjugate due to the symmetrical degeneration of the midbrain structures in PSP (Chen *et al*., 2010). There may be an oblique trajectory, so-called ‘Round the Houses’ or ‘Zig Zag’ signs (Fearon *et al*., 2020).

The slow-velocity vertical saccades can often be difficult to identify in free space. An eye-tracking facility is a valuable tool in these cases. It enables the identification of this subtle early PSP sign because it can examine saccadic velocity and trajectory to a high degree of accuracy. Eye-tracking oculomotor assessment has the advantage over free-space assessment of being recordable, quantitative and objective (Clark *et al*., 2019).

PSP patients make smaller convergence mean amplitudes to symmetrical responses (2.58 ± 1.28°) compared to normal subjects (6.24 ± 1.82°) (Kitthaweesin *et al*., 2002). This convergence issue can present early in the disease (Chen *et al*., 2010) and should be differentiated from primary convergence insufficiency, which is muscular in aetiology as opposed to neural (Ansons and Davis, 2014).

Apraxia of eyelid opening and blepharospasm are two lid signs in PSP which can be challenging to differentiate (Dehaene, 1984). Blepharospasm causes difficulty with lid opening because of activation and contracture of the orbicularis oculi, whereas apraxia of eyelid opening is caused by degenerative supranuclear signals to the levator palpebrae muscles (Hallett *et al*., 2008). The patient may mechanically force the lids open with increased frontalis muscle contraction, which can give the expression of ‘astonishment’ (Phokaewvarangkul and Bhidayasiri, 2019). If the issue is treated effectively with botulinum neurotoxin injection, then blepharospasm is indicated. However, should there be no effect, apraxia of eyelid opening is indicated (Hallett *et al*., 2008). Recent diagnostic criteria (Höglinger *et al*., 2017) group both conditions under the term ‘eyelid opening apraxia’; we adopted this terminology for both conditions here.

Frequent blinking is required to maintain cornea and conjunctival health by removing irritants and spreading the tear film. Reduced spontaneous blink rate is common in PSP (Bologna *et al*., 2009). Reddy et al. (2013) found PSP patients to blink 1.9 times per minute compared to 12.4 blinks per minute with a control group. This gives the appearance of the patient ‘staring’.

Square wave jerks (SWJ) are a type of saccadic intrusion which are common in a typical population with a mean amplitude of 0.7° and frequency of 11.5/min (Abadi and Gowen, 2004). In PSP they are larger with a mean amplitude of 1.4° and a higher frequency of 48/min (Otero-Millan *et al*., 2011). Abnormal SWJs are included in PSP diagnostic criteria (Höglinger *et al*., 2017). It should not be used for PSP diagnosis as an isolated sign due to it also being common in PD and MSA. As SWJ movements are small, they can be difficult to detect in free space. Eye-tracking is often required to confirm their presence.

With training and experience, these orthoptic assessments are quick to complete. For patients with PSP health-related quality of life is poor (Calvert *et al*., 2013), and carer burden is high (Schmotz *et al*., 2017). Fabbri et al. (2024) emphasised the need for a shared, integrated approach to caring for PSP patients. The results from the orthoptic assessment will provide valuable information for the neurology diagnosis and help with the differentiation between PD and atypical Parkinsonian disorders: PSP, MSA, CBS, and DLB (Srivanitchapoom *et al*., 2018). We acknowledge that eventually neurology will home in on the most likely atypical Parkinsonian disorder diagnosis. The advantage of orthoptic input is to refine this differentiation before late-stage degeneration. This benefits the patient with improved access to tailored health and social care services, having a positive effect on the patient’s quality of life and the carer’s burden.

Estimated survival time from diagnosis is between 6 and 12 years for patients with PSP (Williams and Lees, 2009). PSP patients require an extensive multidisciplinary team. The professionals involved include neurologists, speech and language therapists, dieticians, physiotherapists, occupational therapists, social workers, or nurse specialists. Having access to the care and support from these professionals at an earlier stage could potentially extend survival time. Compensatory strategies can be offered at a stage when the patient is able to understand the positive effects (prior to cognitive decline). Speech and language therapy and dieticians can help limit the risk of aspiration pneumonia due to dysphagia through advice on swallowing techniques or diet management. Physiotherapists can help limit the risk of falls through providing exercise and balance training. Occupational therapists can help with daily living activities and improve independence through home safety advice/alterations or mobility device training. These are examples of where early diagnosis and intervention can have a big impact on the patient’s quality of life and survival time.

As with any retrospective analysis of clinical notes, there are associated limitations here, one being a restricted number of cases meeting the inclusion criteria. Selection bias was minimised with strict inclusion criteria. We acknowledge quantitative evidence for certain test results was not available, but misclassification of test results, we suspect, had minimal effect here as we collected data from one clinic which has standardised testing procedures. Different timeframes between disease presentation and orthoptic/neurology/neurosurgery assessment would likely affect the reliability of the diagnosis made. As this was a retrospective analysis, this was a variable we could not control and was not studied. We believe this is a variable which should be controlled and studied in future prospective research.

## Conclusion

PSP presents the clinician with a diagnostic challenge, particularly in the early stages. In our 26 cases of atypical Parkinsonian disorder, the suspected diagnoses after initial orthoptic assessment were consistent with the final most-likely neurology/neurosurgery diagnoses in 80.77% of cases. Orthoptic assessment of atypical Parkinsonian patients is therefore clinically effective for ruling PSP in or out of the clinical picture. It enables earlier and more reliable PSP diagnosis, resulting in enhanced access to health and social care services, having a positive effect on the patient’s quality of life. Detection of subtle early PSP oculomotor signs can be further helped through eye-tracking facilities. We conclude that orthoptists have a valuable role to play within the atypical Parkinsonian disorder multidisciplinary team.
